# The blessing of Dimensionality: Feature Selection outperforms functional connectivity-based feature transformation to classify ADHD subjects from EEG patterns of phase synchronisation

**DOI:** 10.1371/journal.pone.0201660

**Published:** 2018-08-16

**Authors:** Ernesto Pereda, Miguel García-Torres, Belén Melián-Batista, Soledad Mañas, Leopoldo Méndez, Julián J. González

**Affiliations:** 1 Electrical Engineering and Bioengineering Group, Department of Industrial Engineering & Instituto Universitario de Neurociencia (IUNE), Universidad de La Laguna, Santa Cruz de Tenerife, Spain; 2 Lab. of Cognitive and Computational Neuroscience, CTB, UPM, Madrid, Spain; 3 Dept. of Data Analysis, Faculty of Psychological and Educational Sciences, Ghent, Belgium; 4 Division of Computer Science, Universidad Pablo de Olavide, ES-41013 Seville, Spain; 5 Department of Informatics and Systems Engineering, University of La Laguna, Santa Cruz de Tenerife, Spain; 6 Unit of Clinical Neurophysiology, Teaching Hospital Ntra. Sra. de La Candelaria, Santa Cruz de Tenerife, Spain; 7 Department of Basic Medical Sciences, University of La Laguna, Santa Cruz de Tenerife, Spain; University of Craiova, ROMANIA

## Abstract

Functional connectivity (FC) characterizes brain activity from a multivariate set of N brain signals by means of an *NxN* matrix *A*, whose elements estimate the dependence within each possible pair of signals. Such matrix can be used as a feature vector for (un)supervised subject classification. Yet if *N* is large, *A* is highly dimensional. Little is known on the effect that different strategies to reduce its dimensionality may have on its classification ability. Here, we apply different machine learning algorithms to classify 33 children (age [6-14 years]) into two groups (healthy controls and Attention Deficit Hyperactivity Disorder patients) using EEG FC patterns obtained from two phase synchronisation indices. We found that the classification is highly successful (around 95%) if the whole matrix *A* is taken into account, and the relevant features are selected using machine learning methods. However, if FC algorithms are applied instead to transform *A* into a lower dimensionality matrix, the classification rate drops to less than 80%. We conclude that, for the purpose of pattern classification, the relevant features should be selected among the elements of *A* by using appropriate machine learning algorithms.

## 1 Introduction

Machine learning algorithms, and their approach to data mining ranging from pattern recognition to classification, provide relevant tools for the analysis of neuroimaging data (see [1–12] for recent reviews and examples with different neuroimaging modalities and pathologies). Indeed, modern technologies such as magnetic resonance imaging (MRI), magnetoencephalography (MEG) and electroencephalography (EEG) generate an enormous amount of data per subject in a single recording session, which call for exactly these kind of algorithms to extract relevant information for applications such as, e.g., categorical discrimination of patients from matched healthy controls or prediction of individual (clinical and non clinical) variables. A salient feature of all these neuroimaging modalities, however, is that (specially in the case of MRI) the number of features, *p* (anatomical voxels for MRI) is huge (of the order of many thousands), whereas the number of subjects *n* is normally small (typically, two orders of magnitude smaller. See [13] for a recent review). This problem, which is a well-known issue in practical machine learning applications, is termed the *small-n-large-p* effect [14], which aggravates the *curse of dimensionality* associated to this data. Indeed, singling out the (possibly few) relevant features from the many thousands available has been compared to finding a needle in a haystack [7].

In the case of MRI, one way of tackling this issue consists in defining the so-called *regions of interest* (ROIs), an approach whereby the many voxels of the MRI are grouped to produce atlases, i.e., a coarser parcellation of the brain image. ROIs can be defined *ad hoc* or using some criterion such as cytoarchitectonics [15] (structure and organization of the neurons), as it is the case for the classical Broadmann areas of the cerebral cortex. In the case of MEG (and specially of the EEG), this problem is not so serious. In these two neuroimaging modalities, the number of recording sites (sensors for the MEG, or electrodes for the EEG) reduces to at most a few hundred, which, although still large and normally higher than the number of subjects, it is an order of magnitude lower than for the case of the MRI. Besides, and contrary to MRI, M/EEG present the advantage of a much higher temporal resolution (of the order of milliseconds), which allows characterizing diseases where one of the relevant features is the impairment of brain oscillatory activity at frequencies > 1 Hz. Thus, it may seem appealing to turn to these two modalities, where the curse of dimensionality is somehow controlled, for machine learning applications.

Recently, the study of brain activity from M/EEG has benefited from the development of new multivariate analysis techniques that characterizes the degree of functional (FC) and/or effective brain connectivity between two neurological time series (see [16, 17] for reviews). The application of these new techniques entails a paradigm shift, in which cognitive functions are no longer associated to specific brain areas, but to networks of interrelated, synchronously activated areas, networks that may vary dynamically to meet different cognitive demands [18, 19]. The interest of this approach has been confirmed by many studies, which have found that these brain networks are disrupted in many neurological diseases as compared to the healthy state [20–22]. Thus, it is not surprising that machine learning algorithms have been recently combined with M/EEG connectivity analysis to classify subjects as healthy controls or patients suffering from different diseases such as Alzheimer’s [8], epilepsy [10, 11] and Attention Deficit Hyperactivity Disorder (ADHD) [2], and to identify EEG segments with the subjects that generate them [23].

However promising this combination may be, the problem with it is that the number of features is no longer bounded by that of sensors, but instead, for *N* recording sites (whether sensor or electrodes), one has $O(N^{2})$ features, which leads us back to the *small-n-large-p* pathway. Therefore, should we want to use machine learning algorithms for M/EEG connectivity patterns, it is almost compulsory to apply some type of algorithm, such as feature selection, which reduces the dimensionality of the problem. Yet such reduction can be carried out following different strategies. Indeed, there are two main options. One can, on the one hand, using some truly multivariate method to the connectivity patterns to reduce the dimensionality of the feature vector. In this way, features are not actually selected, but mapped to a lower dimensional space using a suitable transformation. On the other hand, features can be selected by means of a machine learning algorithm, which favours those features most relevant for classification.

In this work we apply a well-known machine learning algorithm, the Bayesian Network Classifiers [24] (BNC) to classify 33 children into two different groups (healthy controls and ADHD) from their functional brain connectivity EEG patterns, obtained by using two indices of phase synchronisation (PS). ADHD is a well-known disorder, which has received a lot of attention recently in this framework [13, 25, 26]. Normally, the theta/beta power spectral ratio is used as the (already FDA supported) biomarker of reference to be used as adjunct to clinical assessment of such disease, although the latest literature [13] indicates that things may not be so clear-cut. Besides, little is known [2, 13, 25] about the possibility of using EEG-based FC methods for this purpose (and the best strategy thereof).

Therefore, we compare here the results obtained when applying to these subjects the two different approaches for dimensionality reduction mentioned above: one acting *a priori* on the connectivity patterns, whereby we go back to the “original” scenario with one feature per recording site (i.e., to O(N)), and the other one a “traditional” machine learning feature selection algorithm whereby a subset of the $O(N^{2})$ features is selected based on their redundancy. Specifically, we chose the Fast Correlation Based Filter [27] (FCBF), a fast and efficient algorithm capable of capturing non-linear relationships between features, and the population-based Scatter Search (SS) algorithm [28], which uses a reference set composed of high-quality and dispersed solutions that evolves by combining them.

Finally, we also study the influence on the classification accuracy of different strategies to select the data segments.

We aim at finding out which combination of PS indices and strategy for dimensionality reduction of the feature vector is optimal for classification from FC patterns of scalp EEG data for this data set, in the hope that our results may be useful for other researchers applying the same approach to M/EEG data in different pathologies.

## 2 Methods

### 2.1 Subjects and EEG recording

The data set analysed here is a subset of a larger one described elsewhere [2], thus we only provide a brief account of its most important features. Two groups of subjects between 6 and 10 years old were selected for the study. The first one (patient group) consists of 19 boys suffering from ADHD of combined type, (mean age: 8 ± 0.3 y.), recruited from the Pediatric Service (Psychiatric branch) of the Hospital N.S. La Candelaria in Tenerife. Only subjects meeting ICD-10 criteria of Hyperkinetic Disorder [29] or DSM-V criteria of ADHD combined type [30] were included. The second one (control group) consists of 14 boys (mean age: 8.1 ± 0.48 y.) recruited among the children of hospital staff. Inclusion in any group was voluntary and written informed consent of the subject and his parents/guardians was obtained. The Ethical Committees of the University of La Laguna and of the University Hospital N.S. La Candelaria approved the study protocol, which was conducted in accordance with the Declaration of Helsinki.

**Fig 1:**
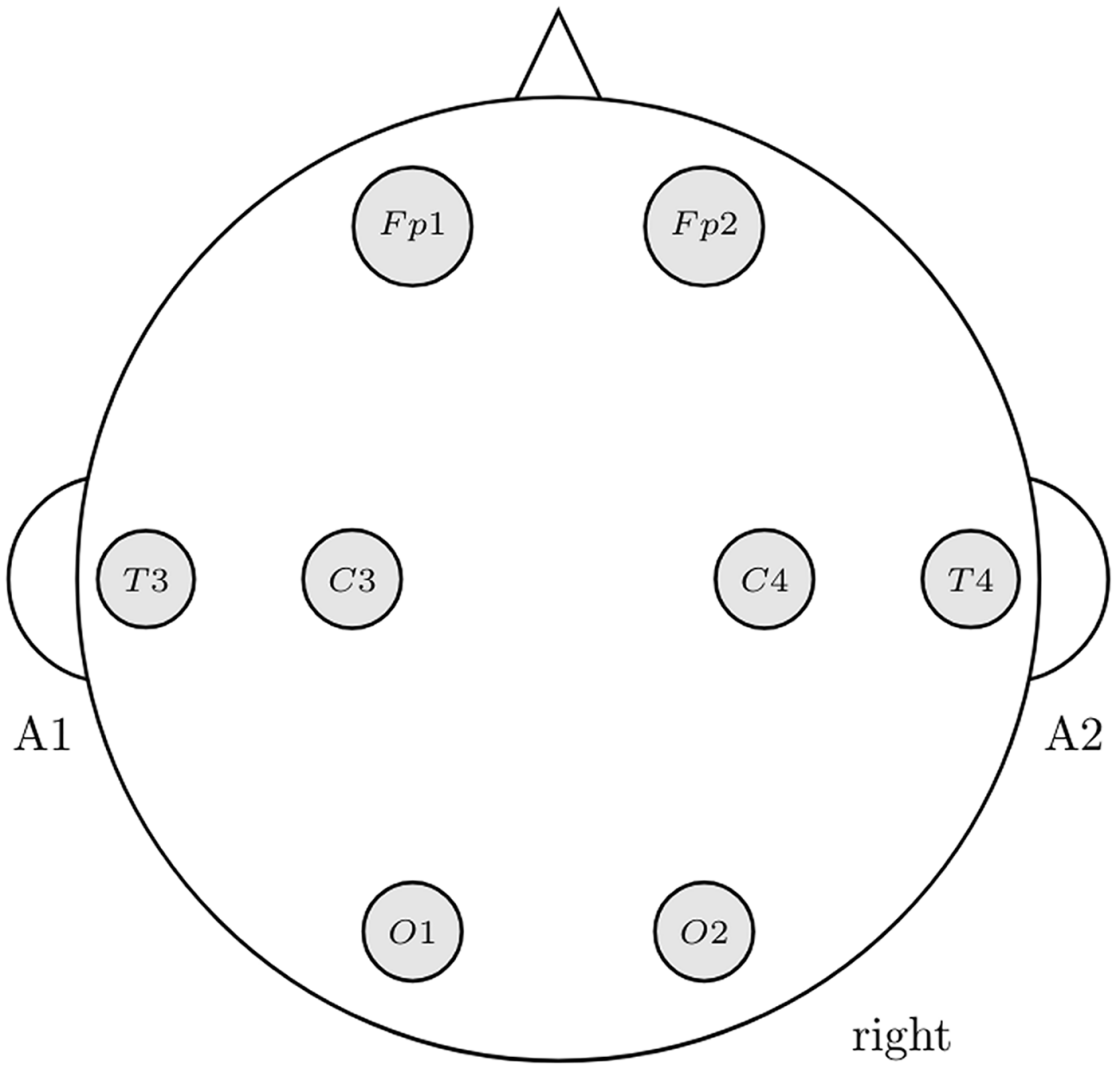
Electrode positions used in our experiments.

EEG recordings lasting approximately one and a half hourwere carried out with the subjects at rest in a soundproof, temperature- and lighting-controlled, and magnetically and electrically shielded room in the clinical neurophysiology service of the hospital. The EEG (sampling rate, 256 Hz) was recorded with open (EO) and closed eyes (EC) between 12:00 and 14:00 using an analogical—digital Nihon Kohden Neurofax EEG-9200 with a channel cap according to the International 10/20 extended system, from the following downsampled set of eight channels (see Fig 1): Fp1, Fp2, C3, C4, T3, T4, O1 and O2. Each electrode was referenced to the contralateral ear lobe. Its impedance was monitored for each subject with the impedance map of the EEG equipment prior to data collection, and kept within a similar range (3 –5 kΩ). The data were filtered online using a high pass (frequency cut-off: 0.05 Hz), a low pass (frequency cut-off: 80 Hz) and a notch filter (50 Hz). Additionally, electro-oculograms and abdominal respiration movements were recorded for artefact detection.

### 2.2 Selection of the data segments

After discarding, by visual inspection, all the segments containing artefacts, the remaining data was divided into non-overlapping segments of 20s. (5, 120 samples), which were detrended and subsequently normalized to zero mean and unit variance. Then, we estimate the stationarity of each segment by calculating the average *ks* statistic of the Kwiatkowski—Phillips—Schmidt—Shin (KPSS) test for stationarity [31], as implemented in the GCCA toolbox [32]. Concretely, for each segment and subject we calculated:

$$\hat{ks^{g}}=\frac{1}{8}\sum_{i=1}^{8}ks_{i}^{g}$$ (1)

where $ks_{i}^{g}$ is the *ks* statistic of electrode *i* for segment *g*. The lower the value of (1), the lower the probability of segment *g* to be trend or mean non-stationary [31]. Therefore, we sorted the values of (1) in ascending order ($\hat{ks^{g_{1}}}<\hat{ks^{g_{2}}}<‥$) and took the five segments *g*_1_, *g*_2_, …, *g*_5_ with the lowest values of the statistic, which are the five most stationary ones among all those available for each subject.

Finally, and prior to the estimation of the FC patterns (see below), the selected data segments were filtered using a Finite Impulsive Response (FIR) filter of zero phase distortion (filter order: 256) in the following five frequency bands: *δ* [0.5 − 3.5*Hz*), *θ* [3.5 − 8*Hz*), *α* [8 − 13*Hz*), *β* [13 − 30*Hz*) and *γ* [30 − 48*Hz*).

### 2.3 Data analysis

#### 2.3.1 Phase synchronisation analysis

Phase synchronisation (PS) refers to a type of synchronized state in which the phases of two variables are locked, whereas their amplitudes are uncorrelated (see [33] for details, and, e.g., [16, 34] for a review of neuroscientific applications). The first step to study PS between two noisy real-valued signal consists of estimating the phases of each signal, which can be done in different ways [35]. We make use here of the approach based on the analytic signal *x*_*a*_(*t*), of a narrow band signal *x*(*t*), which is constructed as follows:

$$x_{a}\left(t\right)=x\left(t\right)+jx_{H}\left(t\right)$$ (2)

where *j* is the imaginary unit ($j=\sqrt{-1}$) and *x*_*H*_(*t*) is the Hilbert transform of *x*(*t*)

$$x_{H}\left(t\right)=\frac{1}{\pi }P.V.\int \frac{x(\tau )}{t-\tau }d\tau$$ (3)

and P.V. stands for principal value. The phase of (2) is:

$$\theta_{x}\left(t\right)=\text{arctan}\frac{x_{H}\left(t\right)}{x(t)}$$ (4)

The relative phase (restricted to the interval [0,2*π*)) between electrodes *i* and *l* is defined as:

$$\varphi_{il}\left(t\right)=\left|\theta_{x_{i}}\left(t\right)-\theta_{x_{l}}\left(t\right)\right|mod2\pi$$ (5)

The most usual way of assessing PS is the so-called *Phase Locking Value (PLV)*, defined as:

$$PLV_{il}=\left|<e^{j\varphi_{il}\left(\tau \right)}>\right|$$ (6)

where < > indicates average, and ∥ the norm of the resulting complex number. By definition, (6) ranges between 0 (no PS, or uniformly distributed *φ*_*il*_) and 1 (complete PS or constant *φ*_*il*_). It is closely related to the well-known coherency function, but taking into account only phase (rather than amplitude) information, and can be estimated very efficiently [36]. A well-known feature of this index when applied to scalp EEG is that it is unable to distinguish true connectivity, which takes place with non-zero time delay [37], from spurious FC between two electrodes recording the activity of a single deep neural source due to *volume conduction*), which is characterized by zero time delay.

Since the existence of a time delay in the interdependence gives rise to a relative phase centred around values other from 0 and *π*. Thus, a variant of (6) has been defined, which is robust against volume conductions effects by ignoring these two relative phase differences [38]:

$$PLI_{il}=|<sign(sin\left(\varphi_{il}\left(t\right)\right)>|$$ (7)

where *sign*(*x*) = 1 if *x* > 0, -1 otherwise. Clearly, (7) is 0 if the distribution of (5) is symmetric around 0 or *π*.

If we were only interested in the patterns of true connectivity with delay between pairs of *N* electrodes, (7) would be the appropriate choice (see, e.g., [39] for a recent example). But for the present application we think that there are good reasons to prefer the combined use of both indices, *PLV*_*il*_ and *PLI*_*il*_. Firstly, it is well-known that indirect, yet neurologically meaningful connections, between two cortical networks via thalamic relay also take place with zero time lag, [40], which make them indistinguishable from volume conduction in this regards (see also [41]). Secondly, from the point of view of characteristic patterns, the fact that PLV is sensitive to the activity of deep brain sources is actually an advantage rather than a problem. Thus, we use both indices (as implemented in the recently released HERMES toolbox [42]) to characterize the patterns of brain dynamics of each subject, as explained in section 2.4.1.

#### 2.3.2 Multivariate surrogate data test

It is well-known that the values of any of the PS indices described in Section 2.3.1, when applied to two finite-size, noisy experimental time series, may be affected by features of the data other than the existence of statistical relationships between them. In order words, one may have, e.g., that *PLV*_*i*, *k*_ > 0 even though *x*_*i*_(*t*) and *x*_*l*_(*t*) are actually independent from each other. To tackle this problem, it is advisable to estimate the significance of the PS indices before applying any classification algorithm. Here, we made use of the (bivariate) surrogate data method [43], whereby the original value of a FC index (say, *PLV*_*il*_ is compared to the distribution of *N*_*s*_ indices calculated from surrogates versions of *x*_*i*_ and *x*_*l*_ that preserve all their individual features (amplitude distribution, power spectrum‥) but are independent by construction. Such *surrogate* signals can be generated in different ways [44, 45]. The simplest strategy in PS analysis consists of estimating the Fourier transform of the signals, add to the phase of each frequency a random quantity drawn from a uniform distribution between 0 and 2*π* and then transform them back to the time domain. In this way, any possible PS between the original signals is destroyed, but, as it turns out, this also destroys any coherent phase relationship present in each individual signal due to the nonlinearity of the system that generates it [44]. Since such nonlinearity cannot be ruled out in the case of EEG data (see, e.g., [46, 47]), more sophisticated algorithms are necessary. Thus, we chose the twin surrogate algorithm [45, 48, 49], which allows to test for phase synchronisation of complex systems in the case of passive experiments in which some of the signals involved may present nonlinear features. This algorithm works on the recurrence plot obtained from the signal, and is parametric, because it requires, for the proper reconstruction of the state space of the systems that generates the data, the embedding dimension *m*, which we estimated by using the false nearest neighbor method [50] and the delay time *τ*, which we took as the first minimum of the mutual information.

In this way, we generated *N*_*s*_ = 99 pairs of surrogate data $\left\{x_{l},x_{i}^{s}\right\}$ (*s* = 1, ‥, 99), and estimated the distribution of *PLV*_*il*_ and *PLI*_*il*_ under the null hypothesis of no PS by calculating the corresponding PS index between each $x_{i}^{s}$ and *x*_*l*_. Finally, the original value of the index (say, PLV) was considered significant, at the p<0.01 level, if $PLV_{il}>PLV_{il}^{s}\;\forall s$.

### 2.4 Classification

#### 2.4.1 Feature vectors

The process of band pass filtering and PS assessment described before gives rise to FC matrices of the following form:

$$A_{R}^{b}=(\begin{array}{ccc} a_{R_{11}}^{b} & a_{R_{12}}^{b} & \ldots \\ a_{R_{21}}^{b} & a_{R_{22}}^{b} & \ldots \\ \vdots & \vdots & \ddots \end{array})$$ (8)

where *R* is either PLV or PLI, *b* = *δ*, *θ*, *α*, *β*, *γ* stands for each of the five frequency bands, and 1 = F3, 2 = C3,…, 8 = O4 are the electrodes as depicted in Fig 1.

Considering that both PS indices are symmetric (i.e., $a_{R_{il}}^{b}=a_{R_{li}}^{b}\;\forall i,l,R$), and that the diagonal values convey no information ($a_{R_{ii}}^{b}\equiv 1\;\forall i$), the total number of features per band and index is *N*_*F*_ = *N*(*N* − 1)/2, where *N* (= 8 in our case) is the number of electrodes analysed, i.e., *N*_*F*_ = 28. In other words, each feature vector per band *b* and index *R* is:

$$\begin{array}{c} A_{R}^{b}=(a_{R_{12}}^{b},\ldots ,a_{R_{18}}^{b},a_{R_{23}}^{b}\ldots ,\\ \;\ldots ,a_{R_{28}}^{b},a_{R_{34}}^{b},\ldots ,a_{R_{68}}^{b},a_{R_{78}}^{b}) \end{array}$$ (9)

**Fig 2:**
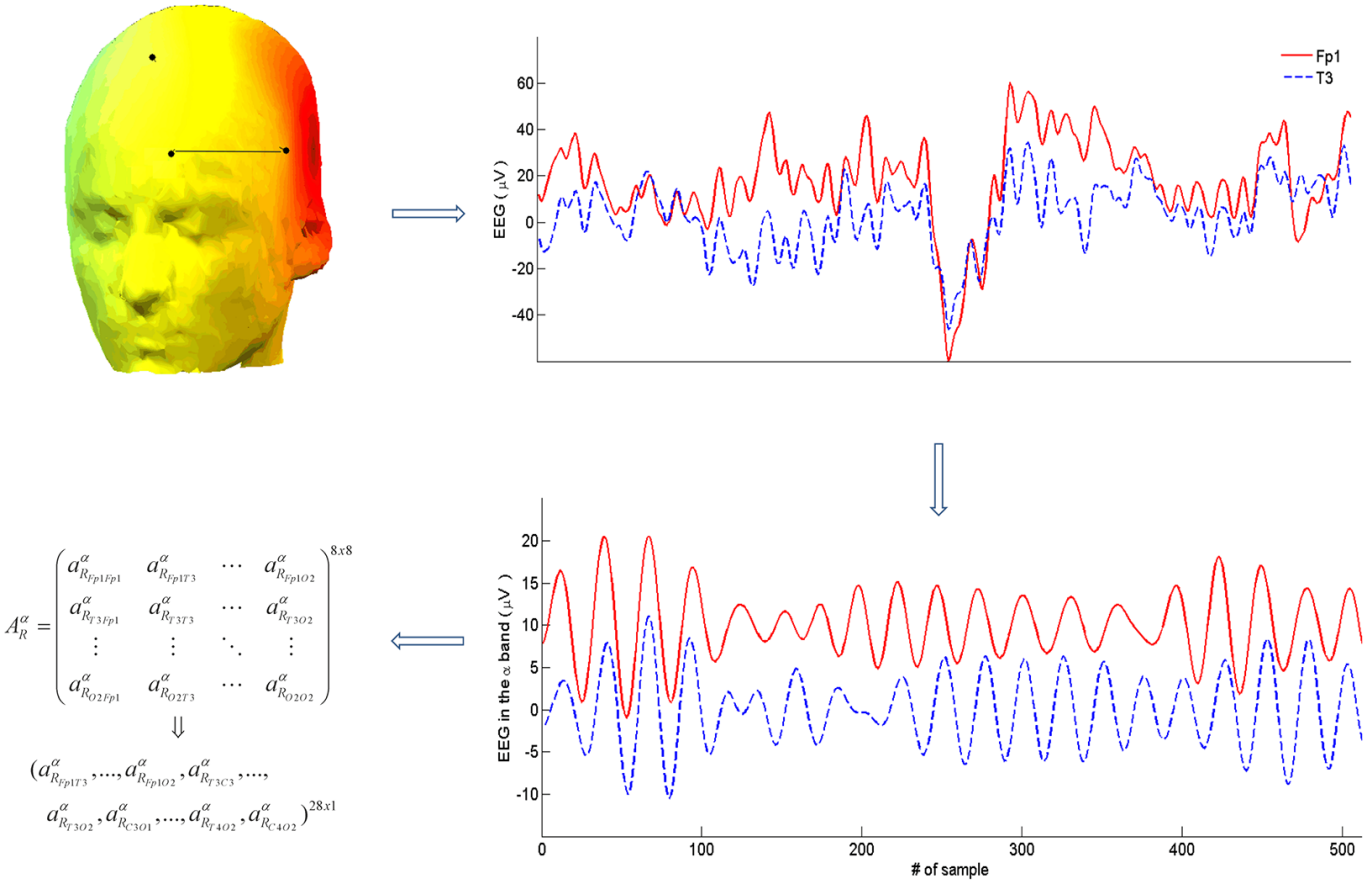
Schematic representation of the construction of the feature vector for each band and index. For each pair of channels as in (a), the raw data in (b) are filtered in the electrodes (Fp1 and T3 in this example), segments such as those in (b) are selected. Then, the signals are filtered in the corresponding frequency bands (e.g., *α* in (c)), and the 8×8 connectivity matrix $A_{R}^{\alpha }$ is obtained, which is finally converted to the 1 × 28 feature vector, after removing the diagonal elements and taking into account the symmetry of both PS indices (i.e., $a_{R_{ii}}^{b}=1;a_{R_{il}}^{b}=a_{R_{li}}^{b}$ ∀*i*, *l*, *b* and *R*).

The whole procedure of feature vector construction is shown in Fig 2 for the case of the *α* band.

If we merge the twenty vectors such as (9) (one per band and condition for both *PLV* and *PLI*), we end up with a feature vector (recently termed as the *FCprofile* [51]) of 28x20 = 560 features:

$$\begin{array}{l} \text{R}=\{A_{PLV}^{O,\delta },A_{PLV}^{O,\theta },\ldots ,A_{PLV}^{O,\gamma },A_{PLI}^{O,\delta },\\ \;\ldots ,A_{PLI}^{O,\gamma },\ldots ,A_{PLV}^{C,\delta },A_{PLV}^{C,\theta },\ldots ,A_{PLI}^{C,\gamma } \end{array}$$ (10)

where the superscripts *O* and *C* stand for open and closed eyes, respectively.

Note that, if one does not apply the surrogate data test, $a_{R_{il}}^{b}>0\;\forall i,l,b,R$ and both conditions. After applying it, however, for a given subject on has $a_{R_{il}}^{b}=0$ for those values of the index *R* that do not pass the test (say, e.g., $a_{PLV_{23}}^{\alpha }$ for open eyes, both but possibly not for closed eyes). Yet this method cannot be used to reduce the dimensionality of (10), because $a_{PLV_{23}}^{\alpha }$ may be indeed significant for another subject. Thus, in general, if we use for the purpose of FC pattern classification, *R* symmetric bivariate indices providing complementary information, calculated on *N* signals in *b* independent frequency bands, we end up with feature vectors such as (10), with *b* × *R* × *N*(*N* − 1)/2 features. These are the feature vectors used by the machine learning classification algorithms described henceforth.

#### 2.4.2 Bayesian Network Classifier (BNC)

In machine learning, classification is the problem of learning a function that identifies the category to which a new observation belongs to. Formally, let T be a set composed by *n* instances described by pairs (**x**_*i*_, *y*_*i*_), where each **x**_*i*_ is a vector described by *d* quantitative features, and its corresponding *y*_*i*_ is a qualitative attribute that stands for the associated class to the vector. The classification problem consists of inducing a function C:X→Y called classifier such that maps from a vector **X** to class labels Y.

We use the BNC [24] due to its ability to explain the causal relationships among the features by using the joint probability distribution. These causal relationships allow to model correlation among the features as well as make predictions of the class label Y. A BNC is a probabilistic graphical model that represents the features and their conditional dependencies as a directed acyclic graph (DAG). A node represents a feature and the edges represent conditional dependences between two features.

**Fig 3:**
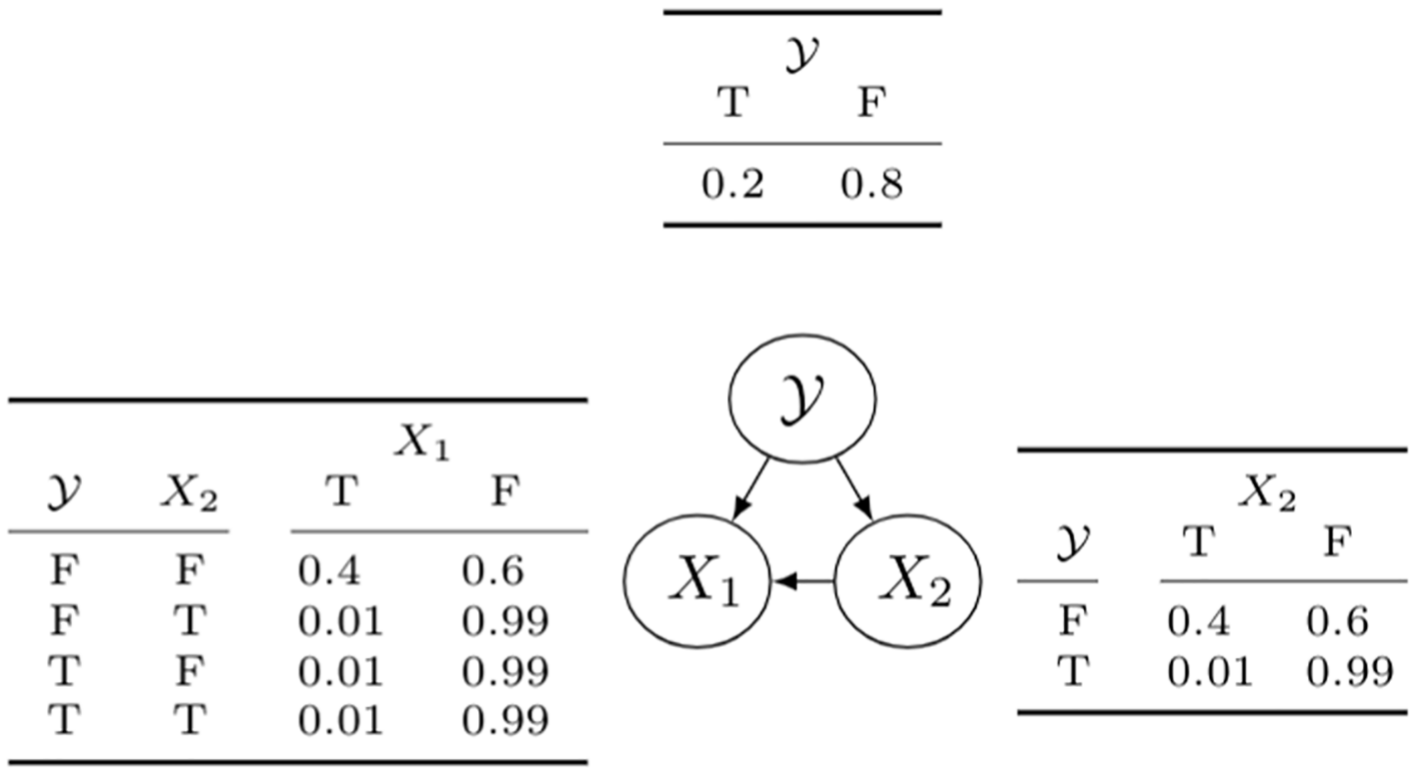
Bayesian network for binary features. Nodes represent features and edges conditional dependencies. The model specifies the conditional Probability Table (CPT) for each feature, which lists the probability that the child node takes on each of its different values for each combination of values of its parents.

Building the classifier consists in learning the structure of the network that best fits the joint distribution of all features given the data, and the set of conditional probability tables (CPTs). Fig 3 shows a Bayesian network for binary data. As we can see, there is always an edge from the class variable Y to each feature *X*_*i*_. The edge from *X*_2_ to *X*_1_ implies that the influence of *X*_1_ on the assessment of the class variable also depends on the value of *X*_2_. Structure learning is computationally very expensive and has been shown to be an NP-hard problem [52], even for approximate solutions [53]. Therefore, learning Bayesian networks normally requires the use of heuristics and approximate algorithms to find a local maximum in the structure space and a score function that evaluates how well a structure matches the data.

To learn the structure of the Bayesian networks, we used the following algorithms: K2 [54], Hill Climbing [55] and LAG Hill Climbing. K2 is a greedy search strategy that begins by assuming that a node has no parents. Then, it adds incrementally that parent, from a given ordering, whose addition increases most the score of the resulting structure. This strategy stops when there is no increase of the score when adding parents to the node. Hill Climbing algorithms begin with an initial network and at each iteration apply single edge operation (adding, deleting and reversing) until reaching a locally optimal network. Unlike K2, the search is not restricted by an ordering of variables. LAG Hill Climbing performs hill climbing with look ahead on a limited set of best scoring steps. With the purpose of quantifying the fitting of the obtained Bayesian networks, we use the Bayesian Dirichlet [56] (BD) scoring function.

#### 2.4.3 Dimensionality reduction

In principle, each of the components of the feature vector 10 offers information on the FC patterns. Thus, we would be faced with the problem of classifying a set of *k* subjects using *n* features, where *k* ≪ *n*. Yet, it is not difficult to foresee that there are cases where there exists a high degree of redundancy between some or many of the such components. For instance, if the connection between the brain networks recorded by electrodes *i* and *l* at a given frequency is direct (or non-existent) then *PLV*_*il*_ and *PLI*_*ij*_ provides essentially the same information. Thus, it is reasonable, to lessen the so-called “curse of dimensionality” to apply some kind of procedure to reduce the number of useful (non-redundant) features to be used for classification. This aim can be accomplished in two different ways. The first one consists of selecting a subset of the available features by using feature selection algorithm from the field of machine learning, which allows maintaining the classification accuracy while minimizing the number of necessary features. The second one, which is specific to multivariate PS analysis, entails the derivation, from each of the matrices 8, of a reduced set of indices that summarize the information of the PS pattern at each frequency band by applying truly multivariate PS methods such as those described, e. g., in [57, 58]. Henceforth, we detail how both approaches were carried out.

*Feature* *selection via machine learning algorithms*

As commented above, in classification tasks the aim of feature selection is to find the best feature subset, from the original set, with the smallest lost in classification accuracy. The goodness of a particular feature subset is evaluated using an objective function, *J*(*S*), where *S* is a feature subset of size |*S*|.

In our experiments we use, as feature selection algorithm, the Fast Correlation Based Filter [27] (FCBF). FCBF is an efficient correlation-based method that performs a relevance and redundancy analysis for selecting a good subset of features. It consist in a backward search strategy that uses Symmetrical Uncertainty (SU) as objective function to calculate dependences of features. Since SU is an entropy based non-linear correlation, it is suitable for detecting non-linear dependencies between features.

By considering each feature as a random variable, the uncertainty about the values of a random variable *X* is measured by its entropy *H*(*X*), which is defined as

$$H\left(X\right)=-\sum_{i}P\left(x_{i}\right){\text{log}}_{2}\left(P\left(x_{i}\right)\right)$$ (11)

Given another random variable *Y*, the conditional entropy *H*(*X*|*y*) measures the uncertainty about the value of *X* given the value of *Y* and is defined as

$$H\left(X|Y\right)=-\sum_{j}P\left(y_{j}\right)\sum_{i}P\left(xi|y_{j}\right){\text{log}}_{2}\left(P\left(x_{i}|y_{j}\right)\right)$$ (12)

where *P*(*y*_*j*_) is the prior probability of the value *y*_*j*_ of *Y*, and *P*(*x*_*i*_|*y*_*j*_) is the posterior probability of a given value *x*_*i*_ of variable *X* given the value of *Y*. Information Gain [59] of a given variable *X* with respect to variable *Y* (IG(Y;X)) measures the reduction in uncertainty about the value of *X* given the value of *Y* and is given by

IG(X|Y)=H(X)-H(X|Y) (13)

Therefore, IG can be used as correlation measure. For example, given the r.v. *X*, *Y* and *Z*, *X* is considered to be more correlated to *Y* than *Z*, if *IG*(*Y*|*X*) > *IG*(*Z*|*X*). IG is a symmetrical measure; which is a desired property for a correlation measure. However it is biased in favor of r.v. with more values and such values have to be normalized to ensure the values have the same scale and so are comparable and have the same effect. To overcome the bias drawback we use the Symmetrical uncertainty (SU) measure; which modifies IG measure by normalizing with their corresponding entropy to compensate the bias

$$SU\left(X,Y\right)=2[\frac{IG(X|Y)}{H(X)+H(Y)}]$$ (14)

SU restricts its values to the range [0, 1]. A value of 1 indicates that knowing the values of either feature completely predicts the values of the other; a value of 0 indicates that *X* and *Y* are independent. So SU can be used as a correlation measure between features.

Based on SU correlation measure, the authors define the approximate Markov blankets as follows.

**Definition 1 (Approximate Markov blanket)** *Given two features* *X*_*i*_ *and* *X*_*j*_ *(i* ≠ *j)* *so that* $SU\left(X_{j},Y\right)\geq SU\left(X_{i},Y\right)$, *then* *X*_*j*_ *forms an approximate Markov blanket for* *X*_*i*_ *iff* $SU\left(X_{i},X_{j}\right)\geq SU\left(X_{i},Y\right)$.

To guarantee that a redundant feature removed in a given step will still find a Markov blanket in any later phase when another redundant feature is removed, they also introduce the concept of predominant feature.

**Definition 2 (Predominant feature)** *Given a set of features* S⊆X, *a feature* *X*_*i*_ *is a predominant feature of S if it does not have any approximate Markov blanket in S*.

**Fig 4:**
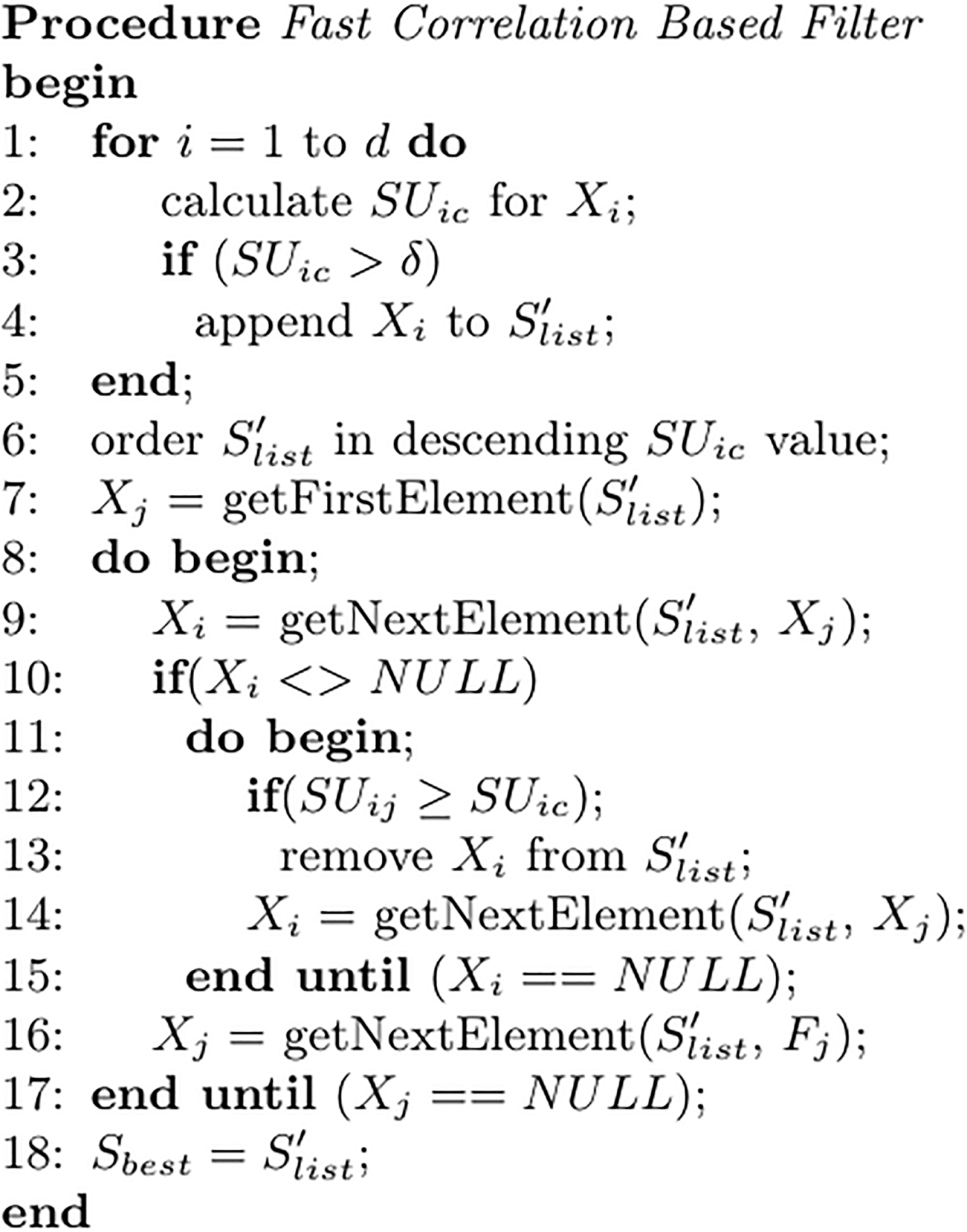
Pseudocode of the Fast Correlation Based Filter algorithm (FCBF).

As we can see in Fig 4, it starts by calculating $SU(X_{i},Y)$ for each feature to estimate the relevance. A feature is considered irrelevant if its value is lower or equal to a given threshold *δ*. In order to detect a subset of predominant features, remaining features are ordered in descending $SU(X_{i},Y)$ value. Then a backward search is performed in the ordered list $S_{list}^{\prime }$ to remove redundant features. The first feature from $S_{list}^{\prime }$ is a predominant feature since it has no approximate Markov blanket. Note that a predominant feature *X*_*j*_ can be used to filter out other features for which *X*_*j*_ forms an approximate Markov blanket. Therefore a feature *X*_*i*_ is removed from $S_{list}^{\prime }$ if *X*_*j*_ forms a Markov blanket for it. The process is repeated until no predominant features are found. In this work we set *δ* = 0 since there is no rule about this parameter tuning and in the datasets under study only a small subset of features have a SU value different to 0.

**Fig 5:**
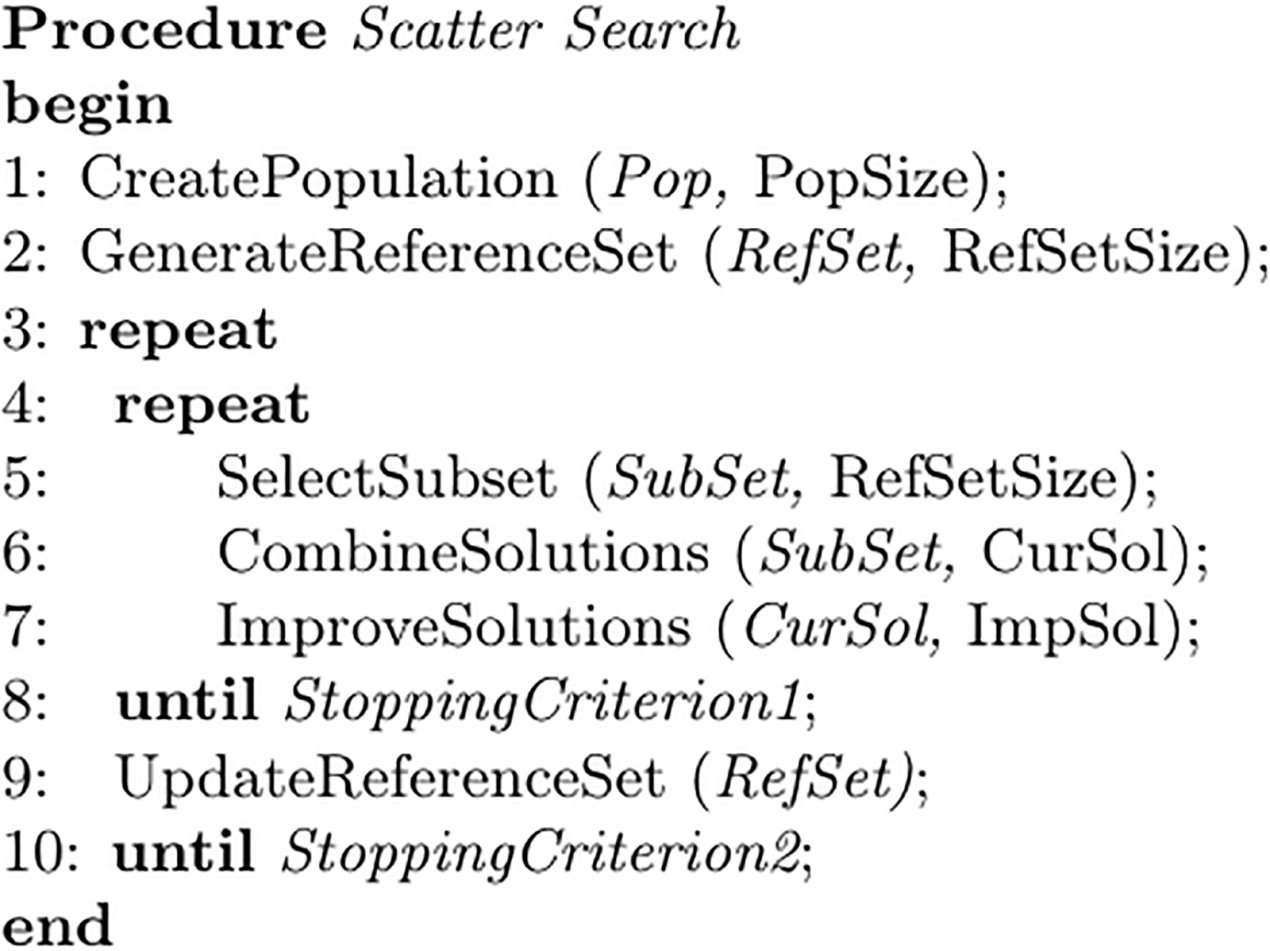
Pseudocode of the Scatter Search algorithm (SS).

The second method we used for feature selection is based on the Scatter Search (SS) metaheuristic proposed by García et al. [28]. SS is a population-based algorithm that makes use of a subset of high quality and dispersed solutions, which are combined to construct new solutions. The pseudocode of SS is summarized in Fig 5. The method generates an initial population of solutions in line 1, which is composed of solutions dispersed in the solution space. In line 2, a reference set of high quality and dispersed solutions is generated from the population. As in standard implementations of SS, the *SelectSubset* method in line 5 selects all subsets consisting of two solutions, which are then combined in line 6. The resulting solutions are then improved in line 7 obtaining new local optima. Finally, a static update of the reference set is carried out in line 9, in which a new reference set is obtained from the union of the original set and all the combined and improved solutions by quality and diversity. For more details, we refer the interested reader to the original paper.

The novelty introduced in this paper is that, in order to measure the quality of the subsets of features selected by the scatter search, we made use of the Correlation Feature Selection (CFS) measure [60] instead of a wrapper approach. CFS evaluates subsets of features taking into account the hypothesis that the good subsets include features highly correlated with the classification, but uncorrelated to each other. It evaluates the worth of a subset of attributes by considering the individual predictive ability of each feature along with the degree of redundancy between them.

The subsest evaluation function of CFS can be stated as follows:

$$M_{S}=\frac{k_{\bar{r_{cf}}}}{\sqrt{k+k\left(k-1\right)\bar{r_{ff}}}},$$ (15)

where *M*_*S*_ is the heuristic merit of a feature subset *S* containing *k* features, $\bar{r_{cf}}$ is the mean feature-class correlation (*f* ∈ *S*), and $\bar{r_{ff}}$ is the average feature-feature inter-correlation.

*Dimensionality reduction using FC methods*: *synchronisation cluster algorithm*

The need for dimensionality reduction in FC studies was already recognized even before the possibility of using them in Machine Learning applications. Indeed, apart from the practical issues associated to the multiple comparison problem (see, e.g., [61] and references therein), it was also demonstrated that weak pairwise correlations (i.e., low values of $a_{R_{ij}}^{b}$) may indicate, rather counter-intuitively, strongly correlated neural network states [62]. In the specific case of PS analysis, Allefeld and co-workers [57] developed a method termed *synchronisation Cluster Analysis* (SCA) whereby the *N* electrodes/sensors from a multivariate M/EEG recording can be considered, under very general conditions, as individual oscillators coupled to a common oscillatory rhythm. The degree of coupling of each oscillator to this global rhythm, *ρ*_*i*_, as well as the overall strength of the joint synchronized behaviour of all the oscillators, *r*, can be inferred from the matrix 8 (see [57] for details). Thus, the FC pattern for each PS index *R* and frequency band *b* comes down to a O(N) vector rather than to a $O(N^{2})$ one. Namely:

$$\rho_{R}^{b}=\left(\rho_{R_{1}}^{b},\rho_{R_{2}}^{b},‥,\rho_{R_{n}}^{b}\right)$$ (16)

Contrary to the approach described above, with SCA we actually do not select a subset of the $O(N^{2})$ features, but rather reduce the number of features to O(N) by taking advantage of the characteristics of the dynamics of the brain synchronisation as described by the data. Note that this approach is also equivalent to another FC methods of dimensionality reduction, such as those based on describing 8 in terms of its *N* eigenvalues [58] or the more recent one based on hyperdimensional geometry [63]. For the 5 frequency bands considered, the two PS indices and the two conditions (open and closed eyes) we have, for each subject, a feature vector *ρ* of N(=8)×5×2×2 = 160 components. Finally, if we take the reduction approach to the limit, and use *r* instead of (16), then the feature vector comprises only 20 components, one for each possible band / index /condition combination.

## 3 Experiments and results

For each subject, we selected three different sets of feature vectors (*R*, *R*^*t*^ and $R_{S}^{t}$), with the aim of determining the influence of each processing steps on classification accuracy. Thus, *R* stands for the feature vectors obtained from 5 segments selected randomly out of all the available one. Then, *R*^*t*^ corresponds to the results from the 5 most stationary segments with the selection procedure described in section 2.2. Finally, $R_{S}^{t}$ is the same that *R*^*t*^ but applying the surrogate data test. Let *ρ* and *r* and *ρ*^*t*^ and *r*^*t*^ be the datasets obtained by applying the SCA algorithm to *R* and *R*^*t*^, respectively. As for $R_{S}^{t}$, there are instances in which the matrix (8) is sparse (i.e., there are many non-significant indices), which prevents the application of the SCA algorithm. Therefore, it was not possible to calculate either $\rho_{S}^{t}$ or $r_{S}^{t}$.

In Table 1, we present the main characteristics of the datasets used in the experiment. The first column refers to the set of feature vector. The following two columns show the datasets obtained from the feature vectors depending on whether SCA was applied or not. Then, for each dataset, the number of features per band is presented and finally, in the last column, we can see the total number of features. Datasets are generated for PLI and PLV phase synchronisation methods. Note that for each band and phase synchronisation method, we include measures from two eyes positions (open and closed).

**Table 1:** Characteristics of the different datasets used in this work for PLI and PLV phase synchronisation methods.

data	FC	dataset	#features/band	#features
R	sca	r	2	10
		*ρ*	16	80
	−	R	28	280
R^*t*^	sca	r^*t*^	2	10
		*ρ*^*t*^	16	80
	−	R^*t*^	28	280
${\text{R}}_{S}^{t}$	−	${\text{R}}_{S}^{t}$	28	280

To evaluate and compare the predictive models learned from data, we used cross-validation; which is a popular method for estimating generalization error based on re-sampling and thus assesses model quality.

In cross-validation, the training and validation sets must cross-over in successive rounds such that each data point has a chance of being validated against. The basic form of cross-validation is *k*-fold cross-validation, which splits the data into k equally sized subsets or folds. Then, *k* iterations of training and validation are performed such that each time a different fold is held-out for validation while remaining *k* − 1 folds are used for learning purpose. Finally, the validation results are averaged over the runs. In general, lower values of *k* produce more pessimistic estimates and higher values more optimistic ones. However, since the *true* generalization error is not usually known, it is not possible to determine whether a given result is an overestimate or underestimate. In spite of this, cross-validation is a suitable estimator for model comparison purposes.

Although cross-validation consumes a great deal of resources, for small sized data the leave-one-out cross-validation (LOOCV) is used since it is an almost unbiased estimator, albeit with high variance. LOOCV is a special case of *k*-fold cross-validation, where *k* equals the number of instances in the data.

All EEG data used in this work are bi-class (e.g., the subjects are either control or ADHD), so that we use sensitivity and specificity scores as performance measures. In our data positive examples refer to label ADHD while negative to control cases. Sensitivity, also called *true positive rate* or recall, measures the proportion of actual positives which are correctly identified as such. Higher values means that higher cases of ADHD are detected. Specificity is the proportion of actual negatives which are identified as such. Higher values correspond to lower probability that a control case be classified as ADHD case.

### 3.1 Baseline classification results

In this section, we analyse the predictive power of the different search strategies for Bayes network structure learning with the datasets under study. Table 2 presents the results. The phase synchronisation method applied is indicated in the first column. Then, the dataset id is shown. The following columns refer to sensitivity and specificity scores for K2, Hill Climbing (HC), and LAG Hill Climbing (LHC). Results with an accuracy higher than 0.7 are in bold.

**Table 2:** Sensitivity and specificity obtained with K2, HC, and LHC search strategies for Bayes network structure learning. Results with accuracy values higher than or equal to 0.70 are marked in bold.

classifier		K2		HC		LHC	
PS	id	sens.	spec.	sens.	spec.	sens.	spec.
PLI	r	0.421	1.000	0.421	1.000	0.421	1.000
	*ρ*	0.421	0.875	0.421	0.813	0.421	0.813
	R	**0.684**	**0.733**	0.684	0.600	0.684	0.600
	r^*t*^	0.947	0.000	0.947	0.000	0.947	0.000
	*ρ*^*t*^	0.684	0.467	0.684	0.467	0.684	0.467
	R^*t*^	0.526	0.467	0.526	0.467	0.526	0.467
	${\text{R}}_{S}^{t}$	1.000	0.000	1.000	0.000	1.000	0.000
PLV	r	1.000	0.000	1.000	0.000	1.000	0.000
	*ρ*	1.000	0.000	1.000	0.000	1.000	0.000
	R	0.579	0.667	*na*	*na*	0.526	0.667
	r^*t*^	0.579	0.000	0.579	0.000	0.579	0.000
	*ρ*^*t*^	0.421	0.733	0.421	0.733	0.421	0.733
	R^*t*^	**0.895**	**0.933**	**0.895**	**0.867**	**0.947**	**0.933**
	${\text{R}}_{S}^{t}$	0.474	0.667	0.474	0.667	0.474	0.667

With the PLI index, K2 achieves an accuracy higher than 0.70 on R dataset. With PLV index, results on *R*^*t*^ dataset achieves a very high accuracy with all search strategies. The other results are lower than 0.70.

### 3.2 Feature selection analysis

Regarding the effect of the feature selection algorithms FCBF and SS on the classification performance, results are shown in Table 3. Those obtained by applying the FCBF are shown in columns 3–8 while those achieved with SS in columns 4–9. As in the baseline scenario, K2 achieves the same accuracy on *R* with PLI index, and all search strategies achieve the best performance scores on *R*^*t*^ using PLV index. With FCBF, the model found by K2 achieves the same accuracy by increasing the sensitivity and decreasing the specificity. The model found by HC improves the accuracy by increasing the sensitivity. Finally, the predictive power found by LHC achieves an accuracy of 100%. This results must be taken with certain caution since they may suggest some kind of overfitting. With SS, the model of K2 improves increases the sensitivity while remaining the specificity value. HC improves in both measures and LHC increases sensitivity reaching a value of 1.

**Table 3:** Sensitivity and specificity obtained with K2, HC, and LHC search strategies for Bayes network strcuture learning after preprocessing with FCBF and SS feature selection algorithms. Results with accuracy values higher than or equal to 0.70 are marked in bold.

PS	id	FCBF						SS					
		K2		HC		LHC		K2		HC		LHC	
		sens.	spec.	sens.	spec.	sens.	spec.	sens.	spec.	sens.	spec.	sens.	spec.
PLI	r	0.421	1.000	0.421	1.000	0.421	1.000	0.421	1.000	0.421	1.000	0.421	1.000
	*ρ*	0.421	0.875	0.421	0.875	0.421	0.875	0.421	0.875	0.421	0.938	0.421	0.875
	R	**0.684**	**0.733**	0.684	0.600	0.684	0.600	**0.684**	**0.733**	0.684	0.600	0.684	0.600
	r^*t*^	0.947	0.000	0.947	0.000	0.947	0.000	0.947	0.000	0.947	0.000	0.947	0.000
	*ρ*^*t*^	0.684	0.467	0.684	0.467	0.684	0.467	0.684	0.467	0.684	0.467	0.684	0.467
	R^*t*^	0.526	0.467	0.526	0.467	*na*	*na*	0.526	0.467	0.526	0.467	*na*	*na*
	${\text{R}}_{S}^{t}$	1.000	0.000	1.000	0.000	1.000	0.000	1.000	0.000	1.000	0.000	1.000	0.000
PLV	r	1.000	0.000	1.000	0.000	1.000	0.000	1.000	0.000	1.000	0.000	1.000	0.000
	*ρ*	1.000	0.000	1.000	0.000	1.000	0.000	1.000	0.000	1.000	0.000	1.000	0.000
	R	0.526	0.533	0.579	0.600	0.632	0.600	0.632	0.533	0.579	0.533	0.684	0.600
	r^*t*^	0.579	0.000	0.579	0.600	0.579	0.000	0.579	0.000	0.579	0.000	0.579	0.000
	*ρ*^*t*^	0.421	0.733	0.421	0.733	0.421	0.733	0.421	0.733	0.421	0.733	0.421	0.733
	R^*t*^	**0.947**	**0.867**	**0.947**	**0.867**	**1.000**	**1.000**	**0.947**	**0.933**	**0.947**	**0.933**	**1.000**	**0.933**
	${\text{R}}_{S}^{t}$	0.474	0.667	0.474	0.667	0.474	0.667	0.474	0.667	0.474	0.667	0.474	0.667

### 3.3 Band relationship analysis

**Fig 6:**
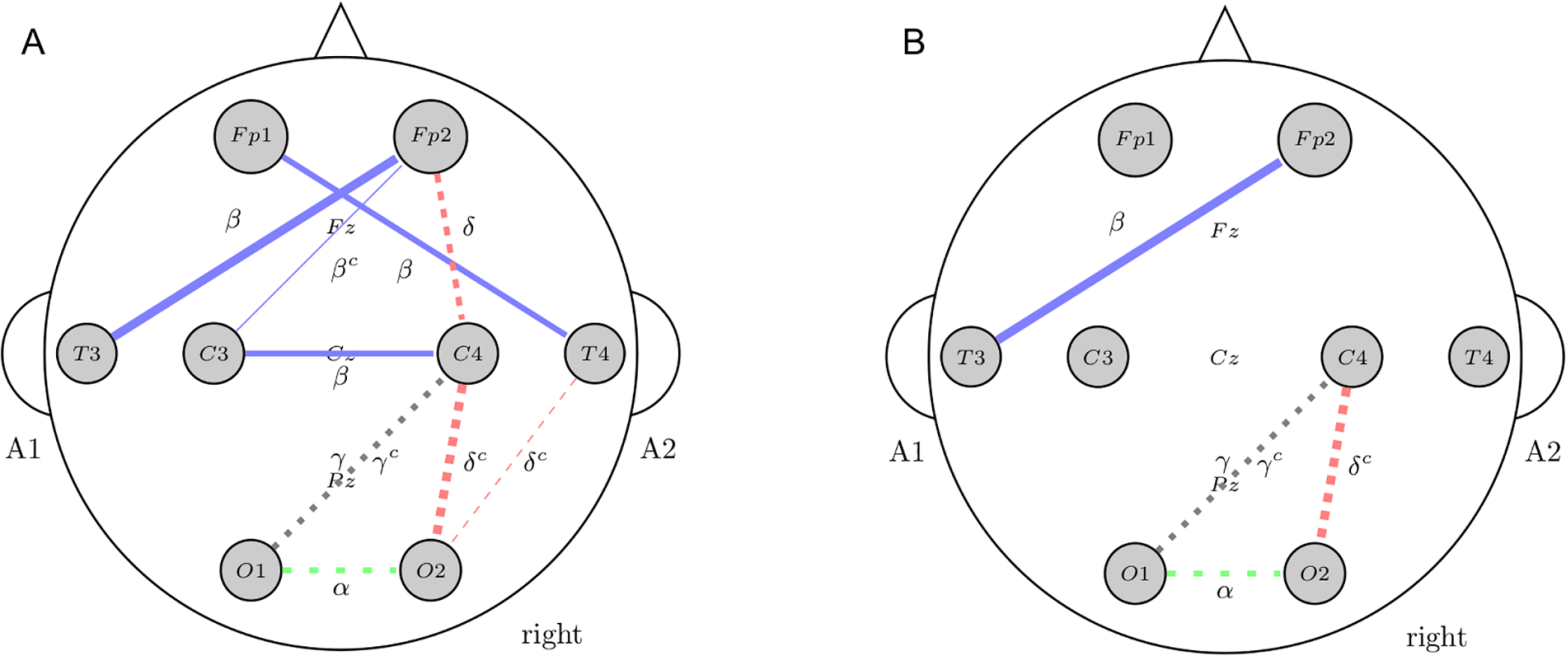
Connections (features) selected by (A) the FCBF and (B) SS feature selection algorithms on *R*^*t*^ dataset with PLV index. The type of line indicates the band (*δ*: dashed; *β*: solid; *α*: loosely dashed; *γ*: dotted), whereas its width is proportional to the correlation of the corresponding connections (see text for details). The superscript ^c^ on the letter for each band indicates the EC condition, whereas connections without superscript correspond to the EO condition.

In this section we analyse the subsets of features selected by FCBF and SS on *R*^*t*^ with PLV. Fig 6 shows the features (connections from now on) selected according to the electrode positions used in our experiments. The connections selected by FCBF are shown in Fig 6A, while those selected by SS are in Fig 6B. The width of the connection is larger for those with higher correlation values with the class label. We used SU as a measure of feature correlation. Superscript *c* stands for connections measured with closed eyes while no superscript refers to open eyes. Along this section we will write the connection between two electrodes E1 and E2 in a given band as (*E*1 − *E*2)_*band*_ for opened eyes and ${\left(E1-E2\right)}_{band}^{c}$ for closed ones.

When applying FCBF, 12 out of the 280 features have non-zeron SU correlation values. Then, the strategy selected 10 of them as predominant features. The selected connections are shown in Fig 6A. The most correlated connections correspond to (*T*3 − *Fp*2)_*β*_ with *SU* = 0.571 and ${\left(O2-C4\right)}_{\gamma }^{c}$ with *SU* = 0.536. Most values are in the range of [0.303 − 0.380] and only two of them have values lower than 0.3. The redundant connections found by this strategy are: ${\left(Fp1-O2\right)}_{\beta }^{c}$ and ${\left(C3-T3\right)}_{\gamma }^{c}$. The earlier one with (*O*1 − *O*2)_*α*_ and the later one with ${\left(O1-C4\right)}_{\gamma }^{c}$.

As we can see in Fig 6B, SS found 5 features that were identified as predominant features by FCBF. It selects the two most correlated connections (*T*3 − *Fp*2)_*β*_ and ${\left(O2-C4\right)}_{\gamma }^{c}$, as well as the features (*O*1 − *C*4)_*γ*_, ${\left(O1-C4\right)}_{\gamma }^{c}$ and ${\left(O2-C4\right)}_{\delta }^{c}$.

**Fig 7:**
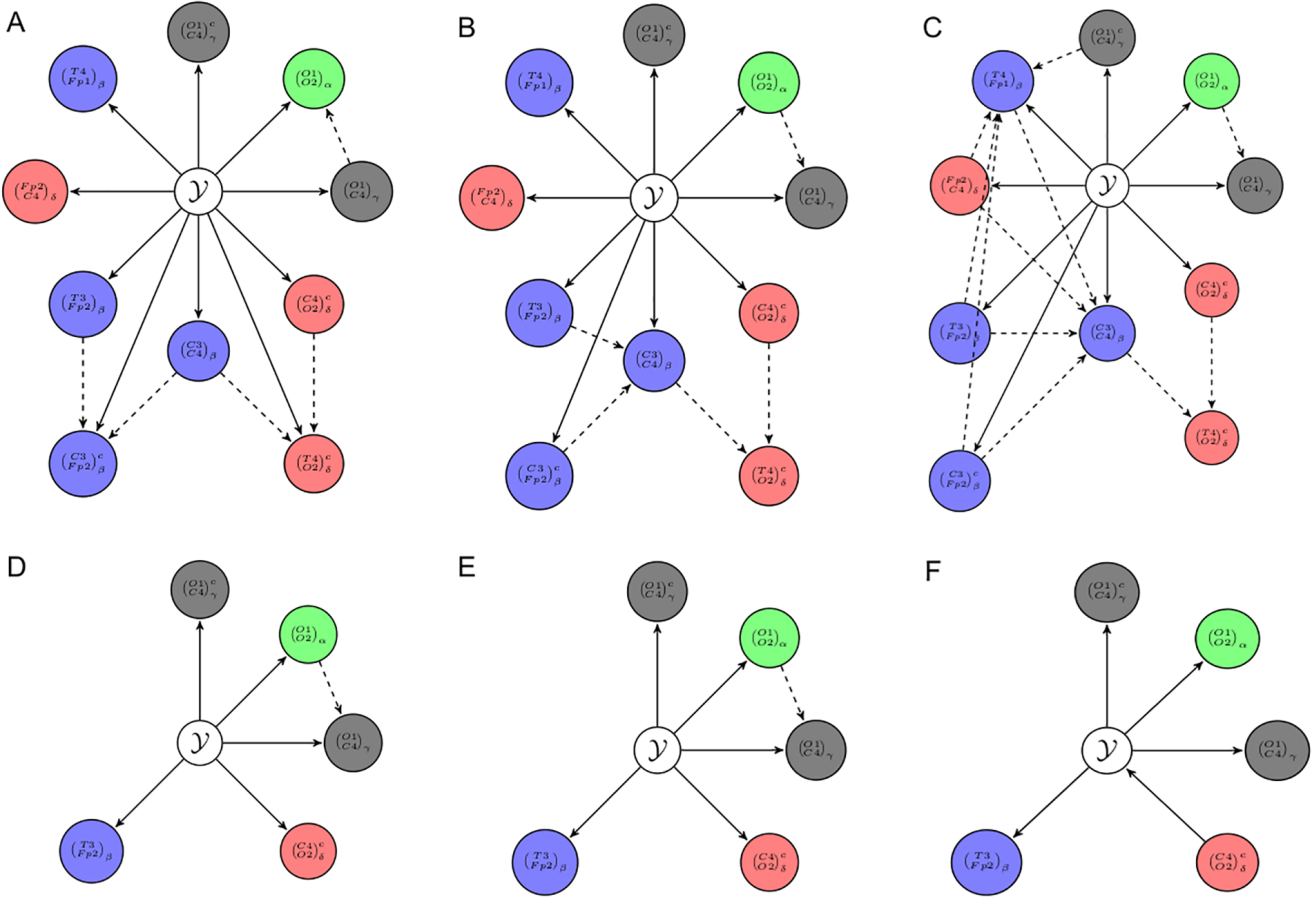
BNC models generated with the connections found by FCBF and SS. Dashed lines represent dependencies between such connections. (A) BNC model generates using K2 strategy with FCBF, (B) HC strategy and FCBF, (C) LHC algorithm and FCBF, (D) K2 and SS, (E) HC and SS, and (F) LHC and SS.

Now we will analyse the BN classifier models generated with the connections selected by FCBF and SS. Fig 7 shows the models obtained using the search methods K2, HC and LHC. As it was explained in this work, in the Bayesian model, edges represent conditional dependencies between the connections. Dashed lines stands for correlations between connections. Due to lack of space, a connection between two electrodes *E*1 and *E*2 in a given band is represented in the figure as ${\left(\begin{array}{c} E1\\ E2 \end{array}\right)}_{band}$ for opened eyes cases and ${\left(\begin{array}{c} E1\\ E2 \end{array}\right)}_{band}^{c}$ for closed ones. We can interpret the generated model as follows.

In Fig 7A we can see the model obtained with K2 with the features selected by FCBF. This model achieves an accuracy of 91.18%. Values of Connection (*O*1 − *C*4)_*γ*_ depend on values of (*O*1 − *O*2)_*α*_. We can also see that connection ${\left(C3-Fp2\right)}_{\beta }^{c}$ receives influence from (*T*3 − *Fp*2)_*β*_ and (*C*3 − *C*4)_*β*_ and ${\left(T4-O2\right)}_{\delta }^{c}$ from ${\left(C4-O2\right)}_{\delta }^{c}$ and (*C*3 − *C*4)_*β*_. It is worth noting that (*C*3 − *C*4)_*β*_ influences two different connections. Fig 7B shows the model with HC, which is quite similar to the previous one and achieves the same accuracy. In contrast to previous model, the dependency between connections (*O*1 − *C*4)_*γ*_ and (*O*1 − *O*2)_*α*_ is inverted. Another difference is that (*C*3 − *C*4)_*β*_ receives influences from (*T*3 − *Fp*2)_*β*_ and ${\left(C3-Fp2\right)}_{\beta }^{c}$. Finally, the model with LHC is presented in Fig 7C. The accuracy is 100% but the complexity of the model has increased considerably and, so, its interpretability. The new dependencies with respect to the previous models are that (*C*3 − *C*4)_*β*_ and (*T*4 − *Fp*1)_*β*_ are influenced by other four connections each. The influence of (*C*3 − *C*4)_*β*_ comes from ${\left(C3-Fp2\right)}_{\beta }^{c}$, (*T*3 − *Fp*2)_*β*_, (*Fp*2 − *C*4)_*δ*_ and (*T*4 − *Fp*1)_*β*_. Finally, (*T*4 − *Fp*1)_*β*_ is influenced by ${\left(O1-C4\right)}_{\gamma }^{c}$, (*Fp*2 − *C*4)_*δ*_, (*T*3 − *Fp*2)_*β*_ and ${\left(C3-Fp2\right)}_{\beta }^{c}$.

Note that in all models generated with connections selected by FCBF, we found that ${\left(T4-O2\right)}_{\delta }^{c}$ depends on connections (*C*3 − *C*4)_*β*_ and ${\left(C4-O2\right)}_{\delta }^{c}$. The dependence between (*O*1 − *C*4)_*γ*_ and (*O*1 − *O*2)_*α*_ is also present in all models although the direction of the arc is different in the first model.

The models obtained with SS are much simpler than those obtained with FCBF, since SS only selected five connections. As we can see in Fig 7D and 7E, K2 and HC algorithms learned the same BN model and it achieves an accuracy of 94.12%. Furthermore, this model shows a single statistical dependence between (*O*1 − *O*2)_*α*_ and (*O*1 − *C*4)_*γ*_. Finally, in Fig 7F, we can see that the model obtained with LHC is slightly different to those obtained previously. It reaches the same accuracy (94.12%), but it presents no dependencies and connection ${\left(C4-O2\right)}_{\delta }^{c}$ is the parent node of Y. Therefore, if Y is known, ${\left(C4-O2\right)}_{\delta }^{c}$ and the other four connections are conditionally independent.

Finally, it is noteworthy that the dependence between (*O*1 − *C*4)_*γ*_ and (*O*1 − *O*2)_*α*_ is also presented in two of the three models generated with the connections selected by SS. This dependence is also found in the models generated previously with FCBF. Thus, we think that this is a robust result.

## 4 Discussion

We have analysed here a topic of great current interest [5, 9], namely the applicability of machine learning algorithms for subject classification from brain connectivity patterns. Concretely, we aimed at elucidating, using data from multichannel human EEG recordings, which is the best strategy to deal with the *curse of dimensionality* inherent to this research approach [14]. For this purpose, we compared different machine learning approaches of feature selection, which pinpoint the optimal subset of features out of all the available ones, with a method (SCA) based on modelling PS in brain dynamics to transform the original features in a reduced set of *new* variables. The whole procedure has been tested in a problem common in this framework, whereby brain connectivity is characterized using bivariate PS indexes between every two electrodes in different frequency bands [16, 19], and this feature vectors is then used to classify subjects in two groups [2, 7, 8, 10]. To the best of our knowledge, this is the first work where such a comparative study has been carried out.

Regarding the results of the classifier, we found that the combination of using the most stationary segments and the PLV yielded a high quality Bayesian network model. Additionally, the original FC features contain more information about the class than the transformed variables *r* and *ρ*. Such features correspond to the most informative connections for classification purposes in the brain connectivity pattern, since irrelevant and redundant ones are removed. Besides, as summarized in Fig 6, the application of FCBF/SS improves the interpretability of the classification model. In fact, Fig 6A and 6B present a very specific frequency/topology pattern of bands and electrodes whose FC, as assessed by PLV, is impaired in the ADHD groups as compared to the healthy one. Thus, low frequency activity in *δ* band is modified in the right hemisphere during CE condition, whereas higher frequency *α*, *β* and *γ* band FC changes mainly in the OE condition for interhemispheric connections. This is consistent with what is known about EEG activity in CE/OE conditions, where low frequency activity is enhanced in the former one, and also with the EEG changes associated to ADHD (see, e.g., [2, 25] and references therein). Note, however, that, as commented before, PLV and PLI measured different things [38], which justifies the use of both of them in the feature vectors. Yet, the best overall performance of any of the algorithms is obtained when only PLV features are considered. PLV is known to be more sensible to volume conduction effects, whereby the activity of a single neural source in the cerebral cortex or beneath is picked up by various electrodes, resulting in EEGs that are correlated. Quite interestingly, and also in line with recent results ([64, 65]), apparently this very reason turns this index into a richer source of information about the characteristic neuroimaging pattern of a given group, and correlates better with the underlying anatomical connectivity [41]. In other words, if one is not interested in the origin of the distinctiveness of the patterns but only wants to generate the most different patterns from two groups, then an index such as PLV, sensitive to changes both in the activity of deep sources and in the interdependence between them, may be more suitable for this purpose than the more robust PLI, which mainly detects the latter changes. Finally, given the inherent multiband nature of EEG changes in ADHD, it may be also interesting to use indices of cross-frequency coupling, which assess the interdependence between different frequencies (see, e.g., [66, 67]) to construct the FC vectors. Another interesting topic for further research would be the dynamic of FC patterns [26]. In both cases, the additional information would come at the price of further increasing the dimensionality, so that the present approach would be even more relevant there.

Another interesting issue that we have investigated is the effect of the segment selection procedure and the estimation of the statistical significance of the FC indices on classification accuracy. The main conclusion in this regard was that the optimal combination consists in selecting the most stationary segments among those available ones (because randomly taking the segments always decreased the accuracy)and at the same time use the values of the indices as such, without any thresholding from the multivariate surrogate data. The result concerning the need for a careful selection of the segments according to their stationarity is hardly surprising. In fact, stationarity is known to be one of the prerequisites to estimate many interdependence indices such as correlation, coherence, mutual information or those based in the concept of generalized synchronisation [16], and the quantitative assessment of the degree of stationarity of M/EEG data segments in functional connectivity applications is receiving increasing attention [68]. Thus, even though stationarity is not a pre-requisite for the application of the Hilbert transform, it is anyhow quite logical that selecting stationary EEG segments that records a single brain state instead of non-stationary ones recording a mixture of them are the best candidates for pattern recognition applications. However, the poorer performance of the surrogate-corrected feature vectors as compared to the “raw” ones is somewhat surprising. Apparently, in the case analysed, the non-significant connectivity indices, whose values are not due to the statistical dependence between the time series but to some feature of the individual data (see, e.g.,[16, 43, 49]), do contain information that is relevant for the classification, so that setting all of them to zero produces more harm than good. Here, again, the message seems to be that the task of dimensionality reduction should be left to machine learning algorithms.

Admittedly, we cannot guarantee that these results will held for other sets of subject / neuroimaging modalities. It may be, for instance, that, contrary to what we have found here, there are instances where the original FC features, even after conveniently selected, do not outperform FC-based methods of dimensionality reduction such as SCA. Or that the use of surrogate data may be useful when the number of electrodes is high. Besides, it may be that BNC could no be always the best classifier. Different algorithms such as SVM [8], linear discriminant analysis [2] or random forest classifiers [10] have proven useful in similar applications. Note, however, that two of these works [8, 10] used previously transformed variables where data reduction is carried out by means of graph theoretic measures, and none of them perform a comparison of different classification algorithms or strategies for dimensionality reduction.

### 4.1 Limitations of the results

A very recent metanalysis of neuroimaging biomarkers in ADHD [13] has warned about the very high accuracies obtained in the literature in these type of studies. There, the small sample sizes and the circularity of the analysis, in which no cross-validation was used in many cases, were pointed out as the main causes of this inflated results. Although we did use cross-validation in the present study, it is clear that the size of our sample is small. Thus, rather that emphasising the absolute values of the accuracies obtained, we stress that they are the changes in this index (i.e., its relative values) after applying different approaches to select the segments and reduce the dimensionality of the feature vector, which represent most interesting outcome of our paper. Furthermore, by sharing all our data and making the code for connectivity analysis publicly available [42, 69], in line with recent efforts from our own research [12], we hope that other labs can apply the proposed classification model as build from our EEGs to their own data. This would be the best check for the validity of the proposed approach by estimating the accuracy of the model in external test sets, or alternatively would allow refining the model by enlarging the sample size. Although issues regarding the different pipelines may still be present, the detailed account we give on the preprocessing steps and automatic segment selection goes online with recent recommendations to improve reproducibility in neuroimaging research [70].

### 4.2 Conclusions

That said, our results suggest that the combination of a careful selection of the data segments, a suitable feature selection method and a machine learning algorithm such as BNC, able to cope with high dimensional data, can turn the curse of dimensionality into a blessing, as the availability of many features allows selecting an optimal subset of meaningful, information-rich variables that can accurately classify subjects from their brain connectivity patterns even from scalp EEG data.

In conclusion, the present outcomes indicate that the use of machine learning algorithms on EEG patterns of FC represents a powerful approach to investigate the underlying mechanisms contributing to changes, as regard to controls, in FC among different scalp electrodes, while allowing at the same time the use of this information for subject classification. They also suggest that this approach may not only be relevant for clinical applications (as it is the case for the theta/beta ratio in ADHD [13]), but also useful to provide insight into the neural correlates of the pathology under investigation.
